# Supplementary material for: HIVprotI: an integrated web based platform for prediction and design of HIV proteins inhibitors
Source: J Cheminform. 2018 Mar 9;10:12. doi: 10.1186/s13321-018-0266-y (PMC5845081; doi:10.1186/s13321-018-0266-y)
Supplement: Supplementary file 2 — Additional file 2. Source code of HIVProtI web server. [file 13321_2018_266_MOESM2_ESM.zip › HIVprotI_Source-code/hivprotI/weka-3-6-6/doc/weka/gui/explorer/Explorer.ExplorerPanel.html]

Explorer.ExplorerPanel


---


|  |  |  |
| --- | --- | --- |
| **Overview** \| **Package** \| **Class** \| **Tree** \| **Deprecated** \| **Index** \| **Help** \| **Weka's home** | |  |
| **PREV CLASS**   **NEXT CLASS** | **FRAMES**    **NO FRAMES**     **All Classes** |  |
| SUMMARY: NESTED \| FIELD \| CONSTR \| METHOD | DETAIL: FIELD \| CONSTR \| METHOD |  |


---


## weka.gui.explorer Interface Explorer.ExplorerPanel

**All Known Implementing Classes:**: AssociationsPanel, AttributeSelectionPanel, ClassifierPanel, ClustererPanel, PreprocessPanel, VisualizePanel

**Enclosing class:**: Explorer

---

``` public static interface Explorer.ExplorerPanel ```

A common interface for panels to be displayed in the Explorer

**Version:**
:   $Revision: 7193 $

**Author:**
:   FracPete (fracpete at waikato dot ac dot nz)

---

| **Method Summary** | |
| --- | --- |
| `Explorer` | `getExplorer()`             returns the parent Explorer frame |
| `java.lang.String` | `getTabTitle()`             Returns the title for the tab in the Explorer |
| `java.lang.String` | `getTabTitleToolTip()`             Returns the tooltip for the tab in the Explorer |
| `void` | `setExplorer(Explorer parent)`             Sets the Explorer to use as parent frame (used for sending notifications about changes in the data) |
| `void` | `setInstances(Instances inst)`             Tells the panel to use a new set of instances. |

**Method Detail**

### setExplorer

```
void setExplorer(Explorer parent)
```

:   Sets the Explorer to use as parent frame (used for sending notifications
    about changes in the data)

    :   **Parameters:**: `parent` - the parent frame

---


### getExplorer

```
Explorer getExplorer()
```

:   returns the parent Explorer frame

    :   **Returns:**: the parent

---


### setInstances

```
void setInstances(Instances inst)
```

:   Tells the panel to use a new set of instances.

    :   **Parameters:**: `inst` - a set of Instances

---


### getTabTitle

```
java.lang.String getTabTitle()
```

:   Returns the title for the tab in the Explorer

    :   **Returns:**: the title of this tab

---


### getTabTitleToolTip

```
java.lang.String getTabTitleToolTip()
```

:   Returns the tooltip for the tab in the Explorer

    :   **Returns:**: the tooltip of this tab


---


|  |  |  |
| --- | --- | --- |
| **Overview** \| **Package** \| **Class** \| **Tree** \| **Deprecated** \| **Index** \| **Help** \| **Weka's home** | |  |
| **PREV CLASS**   **NEXT CLASS** | **FRAMES**    **NO FRAMES**     **All Classes** |  |
| SUMMARY: NESTED \| FIELD \| CONSTR \| METHOD | DETAIL: FIELD \| CONSTR \| METHOD |  |


---
